# Supplementary material for: Effect of Neoadjuvant Therapies on Soft Tissue Sarcomas with Tail-like Lesions: A Multicenter Retrospective Study
Source: Cancers (Basel). 2021 Aug 2;13(15):3901. doi: 10.3390/cancers13153901 (PMC8345724; doi:10.3390/cancers13153901)
Supplement: Supplementary file 1 [file cancers-13-03901-s001.zip › cancers-1303260-supplementary.pdf]

Supplementary Materials

Effect of Neoadjuvant Therapies on Soft Tissue Sarcomas with Tail-Like Lesions: A Multicenter Retrospective Study

Hisaki Aiba, Kunihiro Ikuta, Kunihiro Asanuma, Katsuhisa Kawanami, Satoshi Tsukushi, Akihiko Matsumine, Daisuke Ishimura, Akihito Nagano, Yoji Shido, Eiji Kozawa, Kenji Yamada, Junji Wasa, Hiroaki Kimura, Takao Sakai, Hideki Murakami, Tomohisa Sakai, Tomoki Nakamura and Yoshihiro Nishida

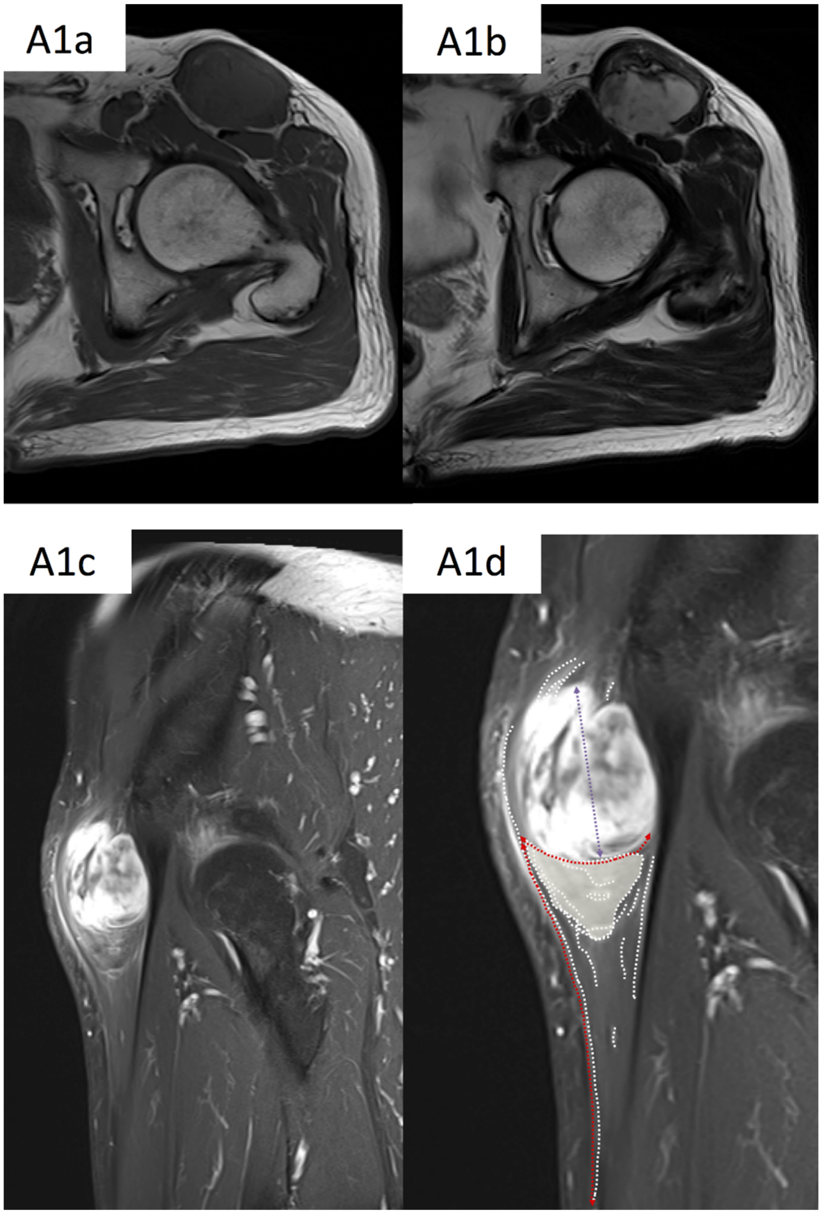

Figure S1. Representative case and the method of measuring the tail-like lesion (deeply located lesion, 65-year-old, male, myxofibrosarcoma, sartorius muscle).

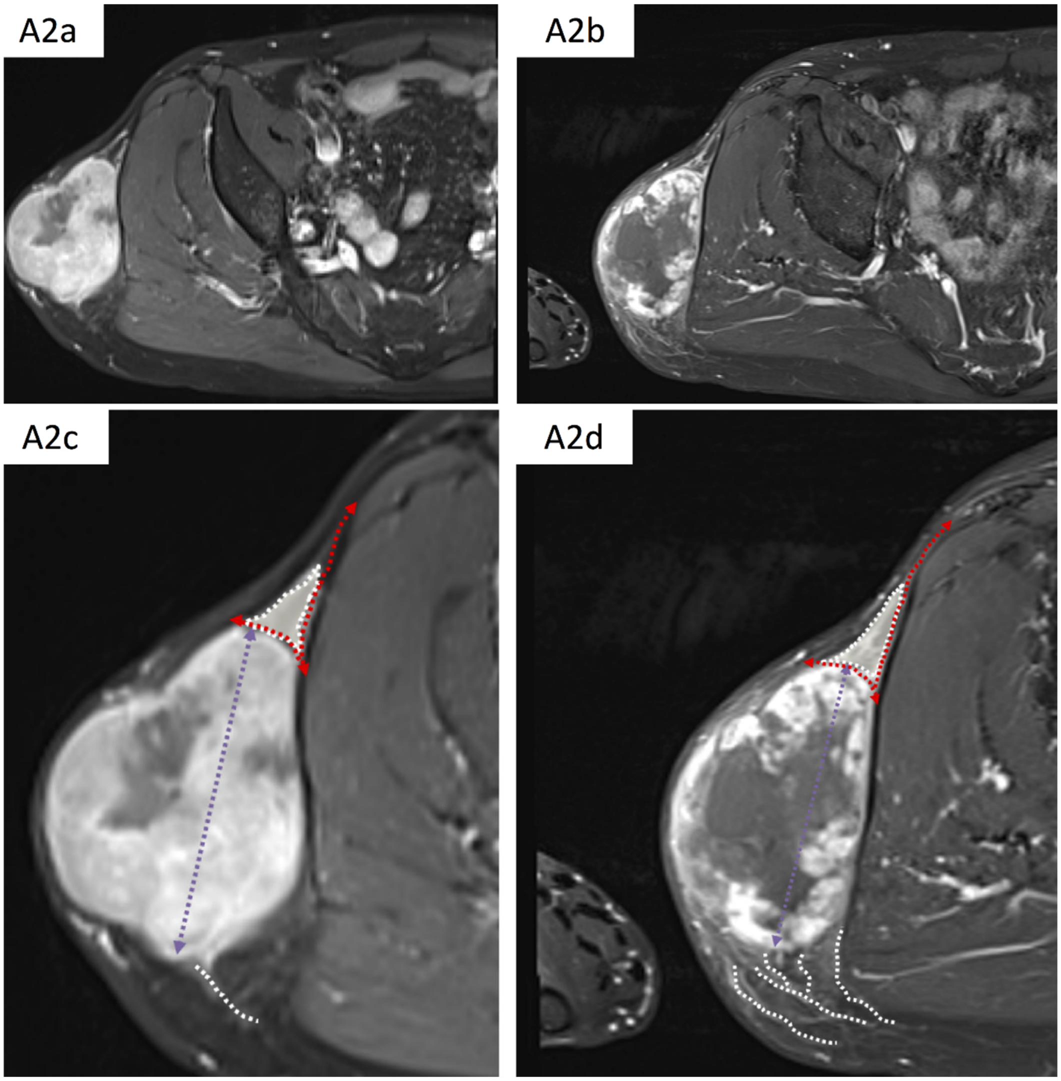

Figure 2. Representative case and the method of measuring the tail-like lesion (superficially located lesion, 59-year-old, male, undifferentiated pleomorphic sarcoma, buttocks).

Table S1. Univariate and multivariate analyses of response to neoadjuvant therapy.

Characteristics (N = 36).		PR (/n)	Univariate[1]	Multivariate[2]
Histology	UPS	1 (/11)	p = 0.21	p = 0.54
	MFS	3 (/13)		
	SS	0 (/4)		
	DDL	2 (/4)		
	Others	2 (/4)		
Length	<5 cm	1 (/7)	p = 0.78	NA
	5–10 cm	5 (/20)		
	>10 cm	2 (/8)		
Location	Lower extremity	5 (/30)	p = 0.03	p = 0.26[3]
	Upper extremity	0 (/3)		
	Trunk	3 (/3)		
Sex	Male	4 (/21)	p = 0.56	NA
	Female	4 (/15)		
Lesion status	Primary	7 (/33)	p = 0.63	NA
	Recurrence	1 (/3)		
FNCLCC grade	Grade 2	1 (/4)	p = 0.89	NA
	Grade 3	7 (/32)		
Biopsy method	Needle	3 (/14)	p = 0.93	NA
	Open	5 (/22)		
Depth	Superficial	2 (/13)	p = 0.46	NA
	Deep	6 (/23)		
Neoadjuvant therapy	RT	0 (/3)	p = 0.56	NA
	Cx	6 (/19)		
	RT + Cx	2 (/14)		

[1] Chi-squared analysis; [2] mutiple logistic regression analysis; [3] analyzed using dummy variables to differentiate between patients with and without lower extremity location. UPS, undifferentiated pleomorphic sarcoma; MFS, myxofibrosarcoma; SS, synovial sarcoma; DDL, de-differentiated liposarcoma; FNCLCC, French Federation of Cancer Centers; RT, radiotherapy; Cx, chemotherapy; NA, not analyzed.

Table S2. Univariate and multivariate analyses of the disappearance of tail-like lesions.

Characteristics (N = 36)		Disappearance of tail-like lesion (/n)	Univariate[1]	Multivariate[2]
**Histology**	UPS	2 (/11)	p = 0.32	NA
	MFS	5 (/13)		
	SS	1 (/4)		
	DDL	1 (/4)		
	Others	1 (/4)		
Length	<5 cm	1 (/7)	p = 0.36	NA
	5–10 cm	8 (/20)		
	>10 cm	3 (/8)		
Location	Lower extremity	8 (/30)	p = 0.04	p = 0.06[3]
	Upper extremity	1 (/3)		
	Trunk	3 (/3)		
Sex	Male	8 (/21)	p = 0.56	NA
	Female	4 (/15)		
Lesion status	Primary	12 (/33)	p = 0.20	p = 0.20
	Recurrence	0 (/3)		
FNCLCC grade	Grade 2	1 (/4)	p = 0.71	NA
	Grade 3	11 (/32)		
Biopsy method	Needle	5 (/14)	p = 0.81	NA
	Open	7 (/22)		
Depth	Superficial	3 (/13)	p = 0.33	NA
	Deep	9 (/23)		
Neoadjuvant therapy	RT	0 (/3)	p = 0.32	NA
	Cx	8 (/19)		
	RT + Cx	4 (/14)		

[1] Chi-squared test; [2] mutiple logistic regression analysis; [3] analyzed using dummy variables to differentiate between patients with and without lower extremity location. UPS, undifferentiated pleomorphic sarcoma; MFS, myxofibrosarcoma; SS, synovial sarcoma; DDL, dedifferentiated liposarcoma; FNCLCC, French Federation of Cancer Centers; RT, radiotherapy; Cx, chemotherapy; NA, not analyzed.

Table S3. Univariate and multivariate analyses of achievement of R0 resection.

Characteristics (N = 36)		R0 resection (/n)	Univariate[1]	Multivariate[2]
**Histology**	UPS	7 (/11)	p = 0.20	p = 0.40
	MFS	8 (/13)		
	SS	4 (/4)		
	DDL	4 (/4)		
	Others	4 (/4)		
Length	<5 cm	5 (/8)	p = 0.63	NA
	5–10 cm	4 (/20)		
	>10 cm	2 (/8)		
Location	Lower extremity	23(/30)	p = 0.15	p = 0.16[3]
	Upper extremity	1 (/3)		
	Trunk	3 (/3)		
Sex	Male	14 (/21)	p = 0.56	NA
	Female	13 (/15)		
Lesion status	Primary	25(/33)	p = 0.73	NA
	Recurrence	2 (/3)		
FNCLCC grade	Grade 2	2 (/4)	p = 0.22	p = 0.57
	Grade 3	25 (/32)		
Biopsy method	Needle	10 (/14)	p = 0.69	NA
	Open	17 (/22)		
Depth	Superficial	9 (/13)	p = 0.55	NA
	Deep	18 (/23)		
Neoadjuvant therapy	RT	2 (/3)	p = 0.01	p = 0.06*[4]
	Cx	18 (/19)		
	RT + Cx	7 (/14)		
Histological response	G1	11 (/15)	p = 0.63	NA
	G2	9 (/13)		
	G3+4	7 (/8)		
RECIST1.1	SD + PD	19 (/28)	p = 0.06	p = 0.09
	PR	8 (/8)		
Disappearance of tail-like lesion	Yes	9 (/12)	p = 1.0	NA
	No	18 (/24)		
Skin reconstruction	Yes	9 (/14)	p = 0.24	p = 0.26
	No	18 (/22)		
Prosthesis use	Yes	2 (/2)	p = 0.40	NA
	No	25 (/34)		
Manipulation of major AVN	Yes	15 (/22)	p = 0.24	p = 0.13
	No	12 (/14)		

[1] Chi-squared test; [2] mutiple logistic regression analysis; [3] analyzed using dummy variables to differentiate between patients with or without lower extremity location. [4] analyzed using dummy variables to differentiate between patients with and without chemoradiotherapy. UPS, undifferentiated pleomorphic sarcoma; MFS, myxofibrosarcoma; SS, synovial sarcoma; DDL, dedifferentiated liposarcoma; FNCLCC, French Federation of Cancer Centers; RT, radiotherapy; Cx, chemotherapy; SD, stable disease; PD, progressive disease; PR, partial response; NA, not analyzed.

Table S4. Univariate analysis of oncologic outcomes.

Characteristics (N = 36)		Univariate[1]	P-value
**Sex (male > female)**	OS	HR = 0.50, 0.12–2.0	p = 0.332
	L-RFS	HR = 1.03, 0.09–11.49	p = 0.978
	D-RFS	HR = 0.49, 0.16–1.48	p = 0.208
FNCLCC grade (grade 2 > 3)	OS	HR = 0.96, 0.12–7.84	p = 0.971
	L-RFS	HR = 0.04, not calculated	p = 0.667
	D-RFS	HR = 0.76, 0.17–3.49	p = 0.743
Depth (superficial > deep)	OS	HR = 0.84, 0.20–3.55	p = 0.813
	L-RFS	HR = 0.69, 0.06–7.63	p = 0.761
	D-RFS	HR = 1.02, 0.07–1.52	p = 0.970
Histological response(grade 3+4 > 1+2)	OS	HR = 0.03, 0.00–32.70	p = 0.331
	L-RFS	HR = 0.02, 0.00–240.53	p = 0.413
	D-RFS	HR = 0.23, 0.03–1.75	p = 0.153
RECIST 1.1 (PR > SD + PD)	OS	HR = 0.38, 0.12–1.23	p = 0.106
	L-RFS	HR = 0.55, 0.05–6.13	p = 0.627
	D-RFS	HR = 0.03, 0.08–6.11	p = 0.198
Disappearance of tail-like lesion (yes > no)	OS	HR = 0.64, 0.13–3.20	p = 0.590
	L-RFS	HR = 1.01, 0.09–11.22	p = 0.992
	D-RFS	HR = 0.34, 0.07–1.52	p = 0.158
Margin status (R0 > R1)	OS	HR = 0.69, 0.16–2.97	p = 0.624
	L-RFS	HR = 0.79, 0.07–8.78	p = 0.847
	D-RFS	HR = 1.24, 0.34–4.51	p = 0.743

[1] Log-rank analysis. Although multiple logistic regression analysis was performed, there were no statistically significant differences. The range indicates the 95% confidence interval of the hazard ratio. HR, hazard ratio; OS, overall survival; L-RFS, local relapse-free survival; D-RFS, distant relapse-free survival; FNCLCC, French Federation of Cancer Centers; SD, stable disease; PD, progressive disease; PR, partial response.

Table S5. Basic characterstics of the patients with or without neoadjuvant therapy.

Characteristics		With Neoadjuvant Therapy (n = 24)	Without Neoadjuvant Therapy (n = 24)	P-value
Histology	UPS	11	8	p = 0.376[1]
	MFS	13	16	
Neoadjuvant therapy	RT	3	0	NA
	Cx	8		
	RT + Cx	13		
Age at diagnosis (mean, SD)		61.8, 10.5	70.8, 14.0	p = 0.014[2]
Tumor length (median, IQR)		76.0, 37.5-113.3	48.0, 34.0–78.0	p = 0.071[2]
Tail-like lesion's length (median, IQR)		30.0, 24.5-48.3	21.5, 18.0-35.0	p = 0.014[2]
Tail-like lesion's thickness (median, IQR)		6.5, 2.2-8.8	4.0, 4.0–11.5	p = 0.076[2]
Location	Lower extremity	20	11	p = 0.024[1]
	Upper extremity	2	8	
	Trunk	2	5	
Sex	Male	16	14	p = 0.551[1]
	Female	8	10	
Lesion status	Primary	22	22	p = 1.0[1]
	Recurrence	2	2	
FNCLCC grade	Grade 1	0	7	p = 0.015[1]
	Grade 2	4	2	
	Grade 3	20	15	
Biopsy method	Needle	12	15	p = 0.344[1]
	Open	12	9	
Depth	Superficial	9	15	p = 0.083[1]
	Deep	15	9	
Surgical margin	R0	15	14	p = 0.768[1]
	R1	9	10	
Adjuvant therapy	Yes	3	12	p = 0.005
	No	21	12	

[1] Chi-squared test; [2] Mann-Whitney U test. UPS, undifferentiated pleomorphic sarcoma; MFS, myxofibrosarcoma; SD, standard deviation; IQR, interquartile range; FNCLCC, French Federation of Cancer Centers; RT, radiotherapy; Cx, chemotherapy; NA, not analyzed.
